# Clinical and volumetric outcomes after vertical ridge augmentation using computer-aided-design/computer-aided manufacturing (CAD/CAM) customized titanium meshes: a pilot study

**DOI:** 10.1186/s12903-020-01205-4

**Published:** 2020-08-05

**Authors:** Alessandro Cucchi, Alessandro Bianchi, Paolo Calamai, Lisa Rinaldi, Francesco Mangano, Elisabetta Vignudelli, Giuseppe Corinaldesi

**Affiliations:** 1 Unit of Oral and Maxillofacial Surgery, Department of Biomedical and Neuromotor Science, University of Bologna, Bologna, Italy; 2 Department of Surgical, Medical, Dental and Morphological Sciences with Interest in Transplant, Oncology and Regenerative Medicine, University of Modena and Reggio Emilia, Reggio Emilia, Italy; 3 Private Practice, Florence, Italy; 4 Department of Prevention and Communal Dentistry, Sechenov First State Medical University, Moscow, Russia

**Keywords:** Alveolar ridge augmentation, Computer-aided-design/computer-aided-manufacturing, Custom-made, Titanium meshes, Dental implants

## Abstract

**Background:**

One of the most recent innovations in bone augmentation surgery is represented by computer-aided-design/computer-aided-manufacturing (CAD/CAM) customized titanium meshes, which can be used to restore vertical bone defects before implant-prosthetic rehabilitations. The aim of this study was to evaluate the effectiveness/reliability of this technique in a consecutive series of cases.

**Methods:**

Ten patients in need of bone augmentation before implant therapy were treated using CAD/CAM customized titanium meshes. A digital workflow was adopted to design virtual meshes on 3D bone models. Then, Direct Metal Laser Sintering (DMLS) technology was used to produce the titanium meshes, and vertical ridge augmentation was performed according to an established surgical protocol. Surgical complications, healing complications, vertical bone gain (VBG), planned bone volume (PBV), lacking bone volume (LBV), regenerated bone volume (RBV), average regeneration rate (RR) and implant success rate were evaluated.

**Results:**

All augmented sites were successfully restored with definitive implant-supported fixed partial dentures. Measurements showed an average VBG of 4.5 ± 1.8 mm at surgical re-entry. Surgical and healing complications occurred in 30% and 10% of cases, respectively. Mean values of PBV, LBV, and RBV were 984, 92, and 892 mm^3^, respectively. The average RR achieved was 89%. All 26 implants were successfully in function after 1 year of follow-up.

**Conclusions:**

The results of this study suggest that the bone augmentation by means of DMLS custom-made titanium meshes can be considered a reliable and effective technique in restoring vertical bone defects.

## Background

Adequate bone volume is an important prerequisite for a predictable, long-term prognosis in implant dentistry and for successful, functional and esthetic implant-supported restorations [1, 2]. Although many studies have confirmed the reliability of short implants to rehabilitate atrophic mandibles and maxillae [1–4], the residual bone volume is often not adequate enough to place dental implants according to criteria of the prosthetically driven implantology, as reported by the most recent guidelines [5]. Thus, the re-establishment of an adequate amount of bone and a proper contour of the alveolar ridge should be considered, in any treatment plan, to allow a prosthetically driven implant placement [6].

Boyne et al. inaugurated the adoption of a titanium mesh for the reconstruction of large discontinuity osseous defects in the jaws [7]. As an alternative to non-resorbable membranes, Ti-meshes exhibit excellent mechanical properties: their rigidity provides extensive space maintenance and prevents contour collapse, their elasticity prevents mucosal compression, and their stability prevents graft displacement [8, 9].

Commercial Ti-mesh plates are designed as planar plates that must be cut and bent in order to be adapted to treat the osseous defect, this procedure is very time-consuming during surgery and considerable skill is required. Additionally, the sharp corners and edges of the plate that have been bent or cut can traumatize the gingiva, perforate the soft tissues and expose the mesh, potentially leading to infections and final failure of the guided bone regeneration (GBR) procedure [10, 11]. With the development of computer-aided-design/computer-aided-manufacturing (CAD/CAM) technologies, accurate pre-operative planning can be established, and surgeons can plan osteotomy and reconstruction procedures, or create patient-specific implants [12]. The advantages of this technique include: restoration of geometrically complex anatomical defects, reduction of operative times, accurate fitting, and eventually performing resection and reconstruction in one step [13–16].

Rapid prototyping based on CAD data, of which one is Direct Metal Laser Sintering (DMLS), has developed rapidly in the last years. DMLS uses a high-powered optic laser to fuse the metal powder to solid components based on a CAD file and creates a 3D object layer by layer. DMLS has many advantages: a wide range of materials, improved functionality, relatively low costs, and the production of ready-to-use near-net-shaped components [12, 17]. Custom-made titanium devices produced for clinical use with the CAD/DMLS technique have been employed in orthopedic surgery, and more recently in oral and maxillofacial surgery [13, 18–25].

The primary aim of this pilot study is to evaluate clinical outcomes of CAD/DMLS customized titanium meshes for alveolar ridge augmentation in terms of surgical and healing complications and volumetric bone gain. Secondary aims are (i) to describe a method for calculation of the regeneration rate, (ii) to measure volumetric outcomes of CAD/DMLS customized titanium meshes, and (iii) to evaluate the effectiveness of osseointegrated standard implants in the augmented sites.

## Methods

### Inclusion and exclusion criteria

Only patients in good health conditions, with adequate oral hygiene, non-smokers or light smokers (≤ 10 cigarettes/day) were considered for enrollment in the present study. Inclusion criteria were the presence of moderate to advanced vertical bone resorption in the posterior areas of the jaws, with impossibility of rehabilitation with prosthetically guided fixed implant-supported metal-ceramic prosthesis due to the lack of bone, and the ability to accept and understand the conditions of the study. Patients were asked to participate for the whole duration of the study, attending the requested follow-up control visits.

The exclusion criteria were: total edentulism; residual bone height < 5 mm; insufficient oral hygiene; heavy smoking habit (> 10 cigarettes/day); abuse of alcohol or drugs; pregnancy; acute local or systemic infection; uncontrolled diabetes or other metabolic disease; immunosuppression-therapy, chemotherapy, or local radiotherapy within the last 5 years. However, therapies for autoimmune disorders and bisphosphonate therapies were not considered as exclusion criteria.

The rehabilitation plan - including the bone augmentation surgery, the implant placement and the implant-supported rehabilitation phase - was discussed with each patient who provided his/her informed written consent. After enrollment, the operators provided an informative sheet with the detailed protocol and the treatment he/she was planned to undergo, respecting Helsinki Declaration guidelines (General Assembly of the World Medical Association 2014). The study was approved by the local Ethics Committee at the University of Bologna (study cod. CMF 01–2017) and all treatments were performed inside the University Dental Clinic.

### The DMLS customized meshes

**Fig. 1:**
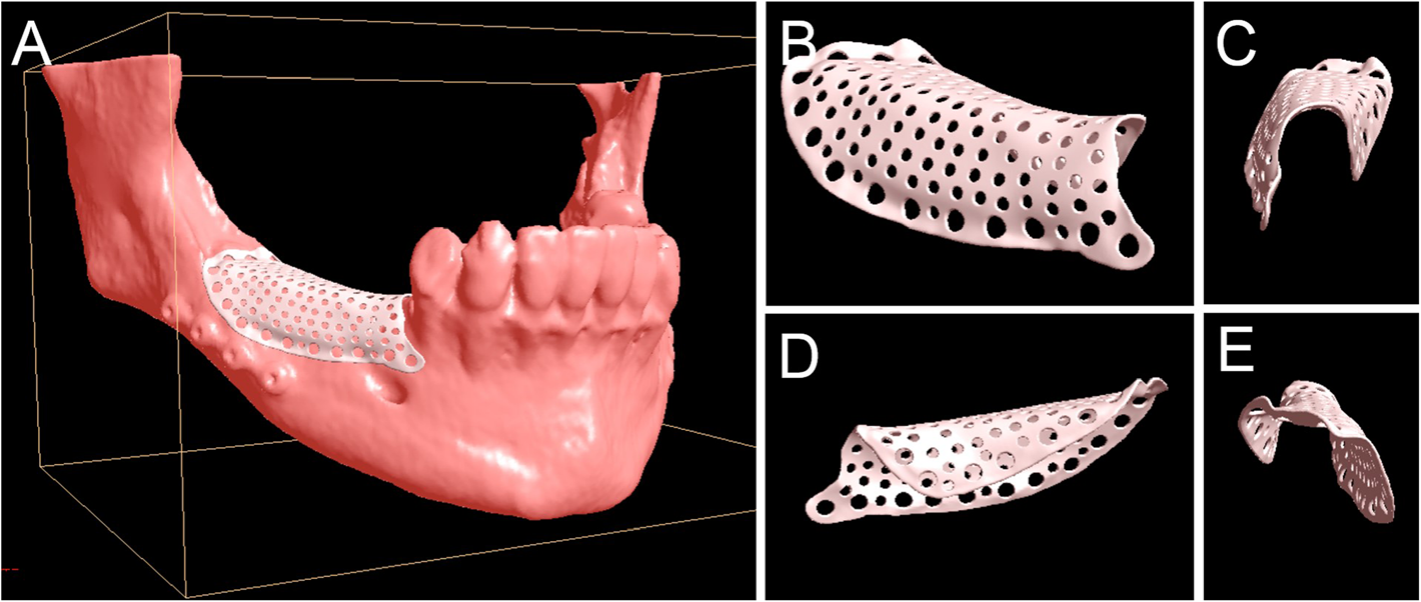
Three-dimensional digital planning of customized mesh for alveolar deficiency in posterior mandible; definitive rendering of the customized mesh. **a** The mesh modelled over the mandible. **b** The mesh alone, buccal view. **c** The mesh alone, anterior (frontal) view. **d** The mesh alone: lingual view. **e** The mesh, posterior view

The workflow for the development of the customized meshes (3D-Mesh®, Biotec Srl, Dueville, Vicenza, Italy) was as follows. The project started from the cone beam computed tomography (CBCT) of the patient. The digital imaging and communication (DICOM) files of the CBCT were sent to the digital specialists of the manufacturer through a dedicated upload area (http://upload.btk.dental/btk3d). Then, the designers reconstructed the bone models in 3D, directly from the CBCT, through segmentation, using a biomedical software (BTK-3D®, Biotec Srl, Dueville, Vicenza, Italy). The virtual bone models of the jaws (maxilla or mandible) were obtained, reproducing the anatomy with particular attention to the area of ​​the bone defect. The mandibular models also included the reproduction of the alveolar inferior nerve; the maxillary models included the alveolar process, the maxillary sinuses, the pterygoid and zygomatic processes. After the bone models were obtained, the area of the bone defect was reconstructed in 3D, using a dedicated CAD software (PLASTYCAD®, 3D COAT, Kiev, Ukraine). Then, the customized mesh was designed over this virtual reconstruction, using the same aforementioned software. The mesh was usually designed in 3D as a perforated texture, with calibrated holes for the insertion of fixation screws and pins (Fig. 1). The project, saved in stereolithographic (STL) format, was shared with the clinician and customized according to his needs, until the final approval. After the final approval, the mesh was produced through selective laser melting (SLM) with a dedicated machine (ProX-DMP100®, 3D System, Rock Hill, SC, USA). This machine was able to build the custom-made mesh, starting from titanium grade 5 micro-powders, layer by layer, using a powerful laser beam (50 W fibre laser with a wavelength of 1070 nm), with layer size of 30 μm. The build envelope capacity of the SLM machine was 100 × 100 × 80 mm. The meshes were usually less than 0.5 mm in thickness with ideal strength and flexibility for the specific clinical application. The meshes were superficially polished through a validated process; then they were decontaminated in an automatic ultrasonic machine, packaged in a cleanroom under controlled atmosphere and sent for sterilization and clinical application. The final object was characterized by high standards of purity and microstructural homogeneity in order to ensure high mechanical performances.

### Operative protocol

All patients received a professional oral hygiene treatment before surgery and were instructed to perform correct oral hygiene procedures for the entire period of the study. The day of surgery (T0), antibiotic prophylaxis (Amoxicillin/Clavulanic Acid 2.0 g) was administered 2 h prior to GBR intervention. The area to be treated was anesthetized using Articaine hydrochloride 4% with epinephrine 1:100.000. The surgery started with a mid-crestal incision which is useful to raise full-thickness buccal and lingual/palatal flaps in order to expose the residual alveolar ridge. The most critical anatomical structures, such as mental nerve or infraorbital nerve, were identified and gently isolated. Subsequently, both flaps were carefully managed to obtain an adequate release for their following coronal advancement. Approximately, 0.5–1.0 g of autogenous bone was harvested from the buccal aspect of adjacent cortical bone using a bone scraper; additionally, the cortex was repeatedly perforated to promote the migration of osteogenic/osteoprogenitor cells under the mesh at the end of surgery. The grafting material was prepared by mixing harvested autologous bone with 50% of xenograft (Zcore®, Osteogenics Biomedical, Lubbock, Texas) and peripherical venous blood. The surgery continued with filling the bone defect with grafting material. Then, the mesh was applied in situ and fixed on the buccal and/or lingual side using two or three titanium mini-screws to achieve a perfect stability. Finally, surgical flaps were coronally advanced and sutured using horizontal mattress for flap overlapping and multiple interrupted sutures for hermetic closure of the wound. Patients were instructed to consume a fluid diet for the first 15 days and a soft diet for the next 15 days.

**Fig. 2:**
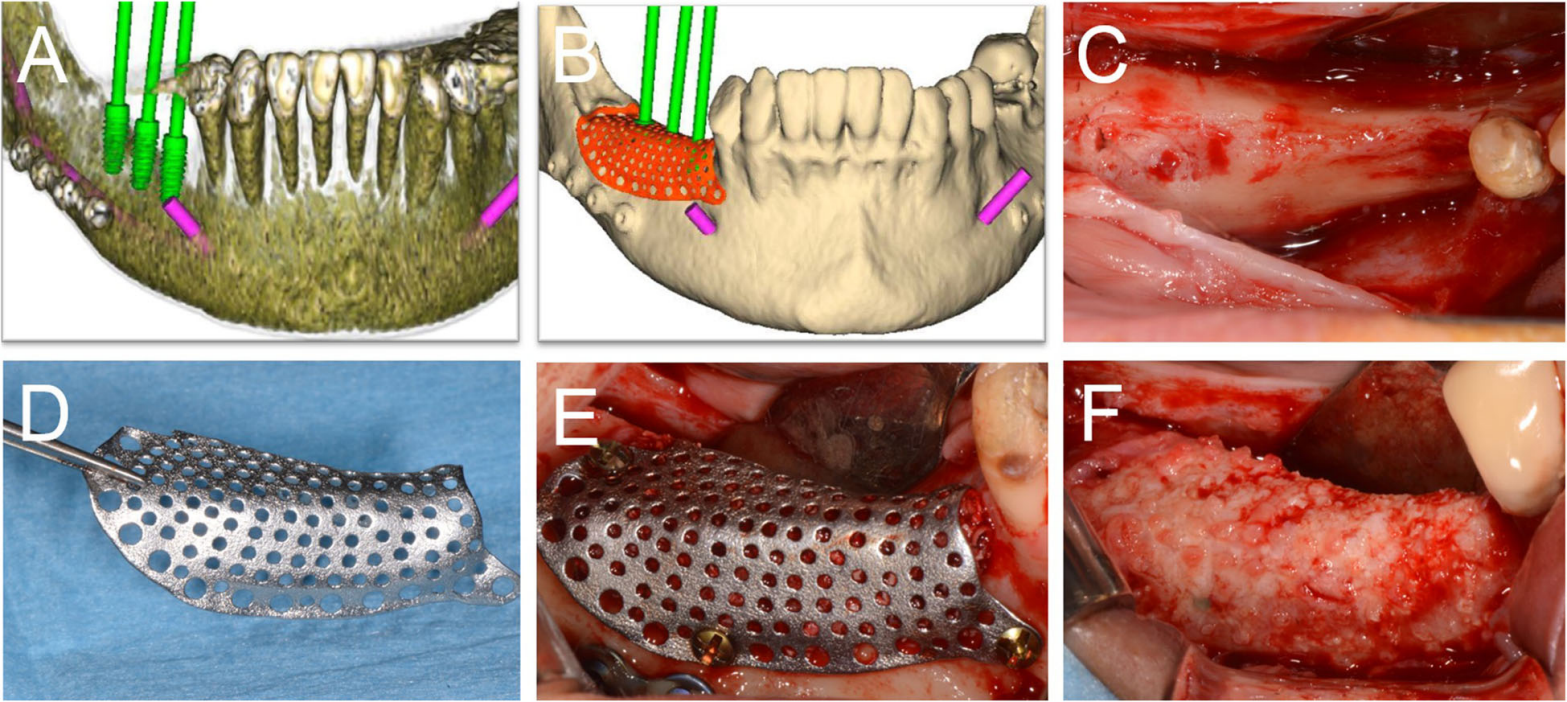
Digital planning of the implants (**a**) and the customized mesh (**b**) in the posterior mandible. **c** Alveolar ridge before augmentation. **d** CAD/CAM mesh. **e** Fixation of the mesh with three titanium screws. **f** Alveolar ridge 6 months after bone augmentation

**Fig. 3:**
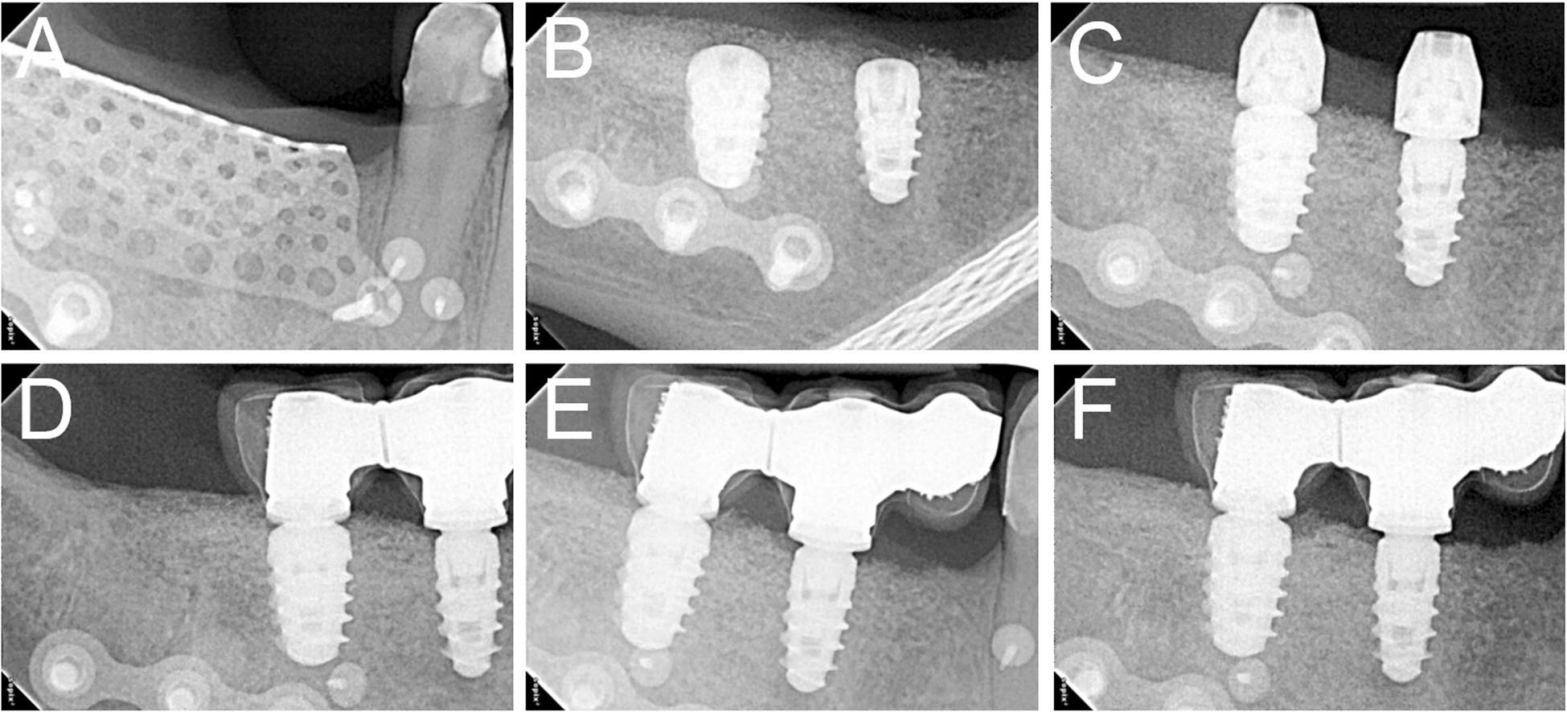
Radiographic images of the customized mesh in position (**a**), of the implants placed in regenerated bone (**b**), the abutments (**c**), and the delivery of the final restoration (**d**). Radiographic controls 6 months (**e**) and 1 year (**f**) after the delivery of final restoration

**Fig. 4:**
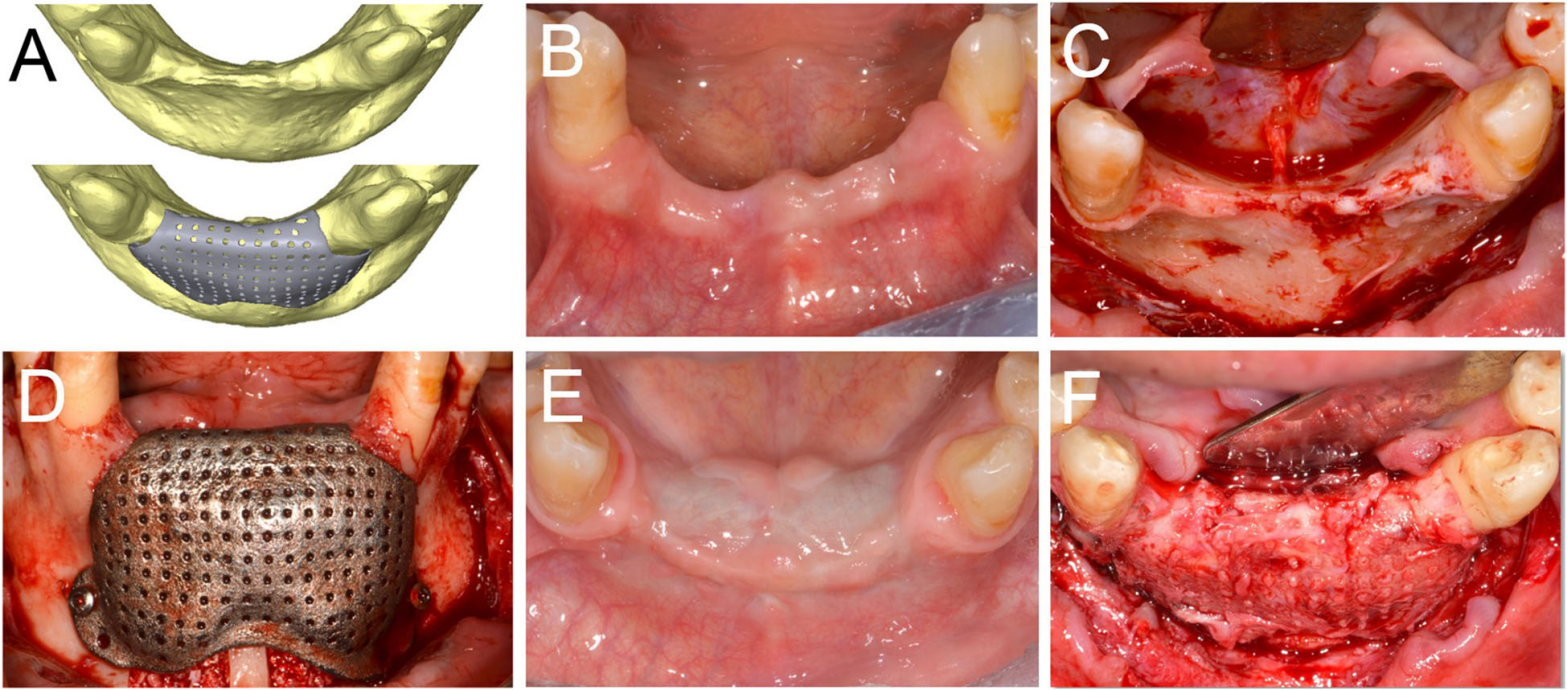
Virtual rendering of customized mesh in anterior mandible (**a**). Soft tissues of the edentulous ridge before bone augmentation (**b**). Alveolar ridge with a severe defect (**c**). Fixation of the CAD/CAM mesh with two titanium screws (**d**). Soft tissues at 6 months, after uneventful healing (**e**). Alveolar ridge 8 months after bone augmentation (**f**)

**Fig. 5:**
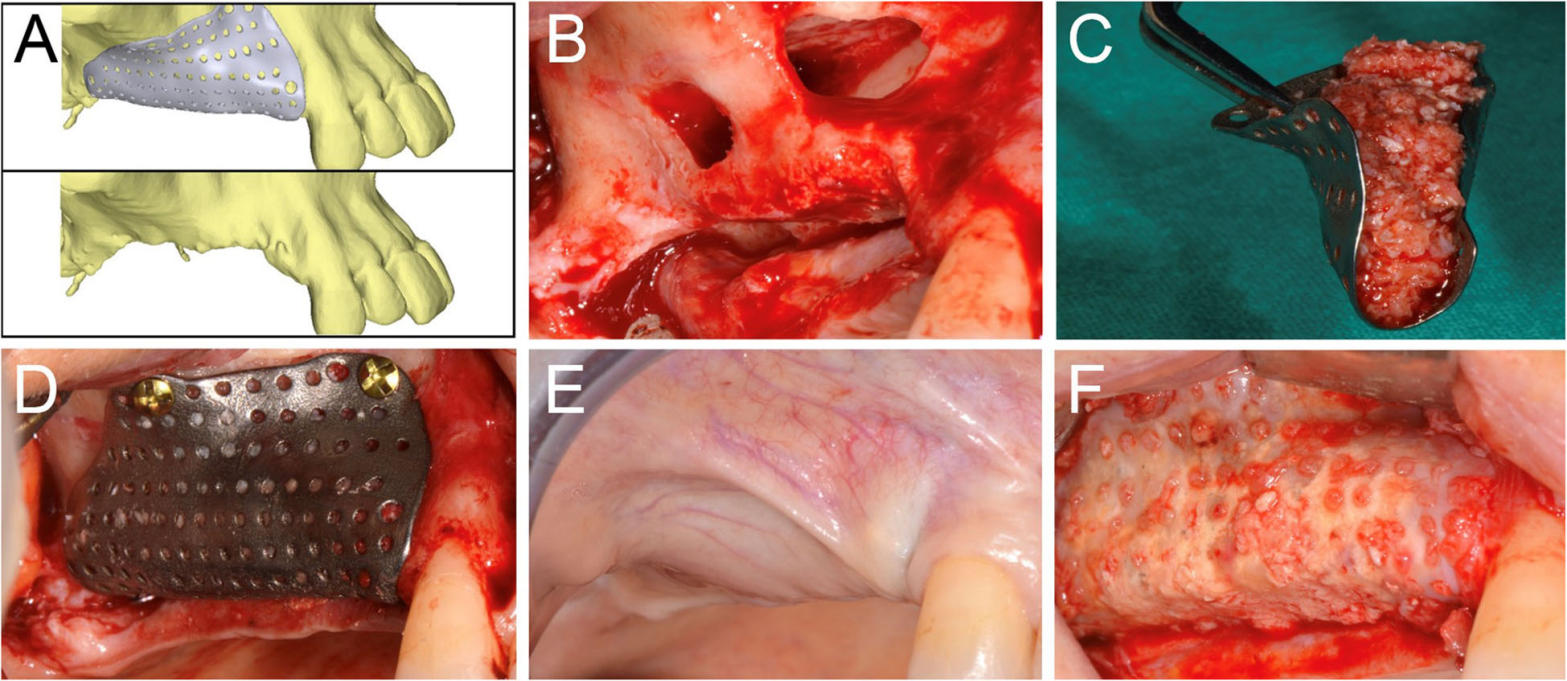
Virtual rendering of customized mesh in posterior maxilla (**a**). Alveolar ridge before augmentation, showing a double bone window for maxillary sinus lift (**b**). CAD/CAM mesh filled with 50:50 of autogenous bone and xenograft material (**c**). Fixation of the mesh with three titanium screws (**d**). Soft tissues at 6 months, after uneventful healing (**e**). Alveolar ridge 9 months after bone augmentation (F)

**Fig. 6:**
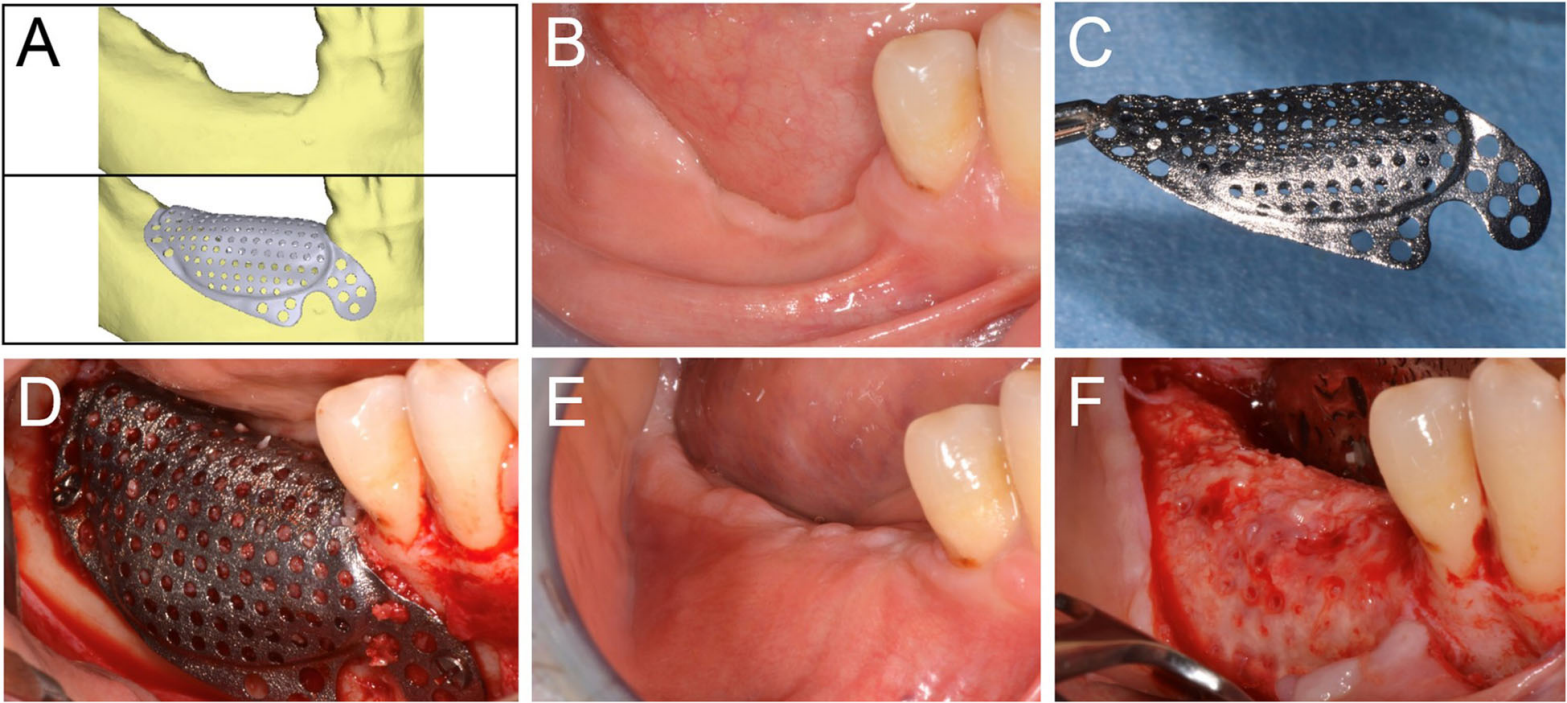
Virtual rendering of customized mesh in the posterior mandible (**a**). Soft tissues of edentulous ridge before bone augmentation (**b**). CAD/CAM mesh (**c**). Fixation of the mesh with three titanium screws (**d**). Soft tissues at 6 months, after uneventful healing (**e**). Alveolar ridge 6 months after bone augmentation (**f**)

After 6-8 months of submerged healing (T1), the augmented sites were re-opened with a crestal linear incision. The removal of the barrier devices and the mini-screws was followed by implant placement: the sites were prepared according to the manufacturer’s protocol, using twisted drills under abundant irrigation. Two or more tapered dental implants (BT Safe®, Biotec Srl, Dueville, Vicenza, Italy), depending on the clinical treatment plan, were placed in the prosthetically ideal position. In some cases, a connective tissue graft was performed to increase the thickness of the peri-implant soft tissues (Figs. 2, 3, 4, 5 and 6).

Antibiotic therapy was prescribed to reduce the risk of infections, both at T0 and T1: Amoxicillin/clavulanic acid at 3 mg/day for 7 days (or clindamycin at 600 mg/day for 6 days in penicillin-allergic patients). We also recommended anti-inflammatory therapy with nimesulide at 200 mg/day for 3 days and 100 mg/day for 3 more days or alternatively ibuprofen at 1800 mg/day for 3 days and 1200 mg/day for 3 more days. After each surgical phase we also recommended daily mouth rinses with chlorhexidine 0.2% for 3 weeks.

The implants were left submerged for a period comprised between 3 and 6 months, depending by the location (for the maxillary implants a submerged healing period of 6 months was requested). After the re-opening surgery, the prosthetic phases started with final impressions, in order implant-supported fixed metal-ceramic restorations could be delivered to the patients 1 or 2 weeks later (T2). All definitive restorations were functionally loaded, with careful reduction of occlusal contacts during lateral and protrusive excursions, to potentially reduce harmful vectorial forces.

### Data collection

At the first visit, personal data, medical and dental history, and patients’ habits were collected by means of self-anamnesis and self-completed written questionnaires. A clinical examination was carried out to assess the presence of chronic or aggressive periodontitis, the presence of parafunctional activities (bruxism or clenching), and the gingival biotype (thick or thin). During surgery and postoperative visits, all clinical and healing data were recorded on a specific data collection form which was updated at every following appointment.

The following parameters were recorded between T0 and T1: quadrant of interest, defect type (horizontal/vertical), periosteum type [26], membrane application, planned bone volume (PBV), surgical complications, and above all healing complications. During the re-entry surgery (T1), the data collection included bone density, pseudo-periosteum type [27], number of implants inserted, implant stability, vertical bone gain (VBG), lacking bone volume (LBV), regenerated bone volume (RBV), and regeneration rate (RR = RBV/PBV.100). Finally, implant and prosthetic success were evaluated during the follow-up period according the to the criteria described by Buser et al. in 1994 [28], and modified by Albrektsson & Zarb in 1998 [29].

### Volumetric analysis

The planned bone volume (PBV) was measured after virtual mesh planning by the manufacturing companies; the reconstructed bone volume (RBV) was obtained by using the previously-established software, starting from the pre- and post-operative cone-beam-computed-tomographies (CBCT); the lacking bone volume (LBV) was calculated as difference between the PBV and RBV; finally, the regeneration rate (RR%) corresponding to the percentage of RBV compared to the PBV.

The volumetric analysis was carried out using three different softwares: software A was used for viewing, processing and analyzing 3D data (Amira®, Zuse, Berlin, Germany); software B was used for modeling, composing and rendering the 3D- and 2D- images (Blender®, Blender Foundation, Amsterdam, Netherlands); and software S was used for the conclusive measurements of the bone volume of the 3D models (Slicer 3D®, Boston, MA, USA).

**Fig. 7:**
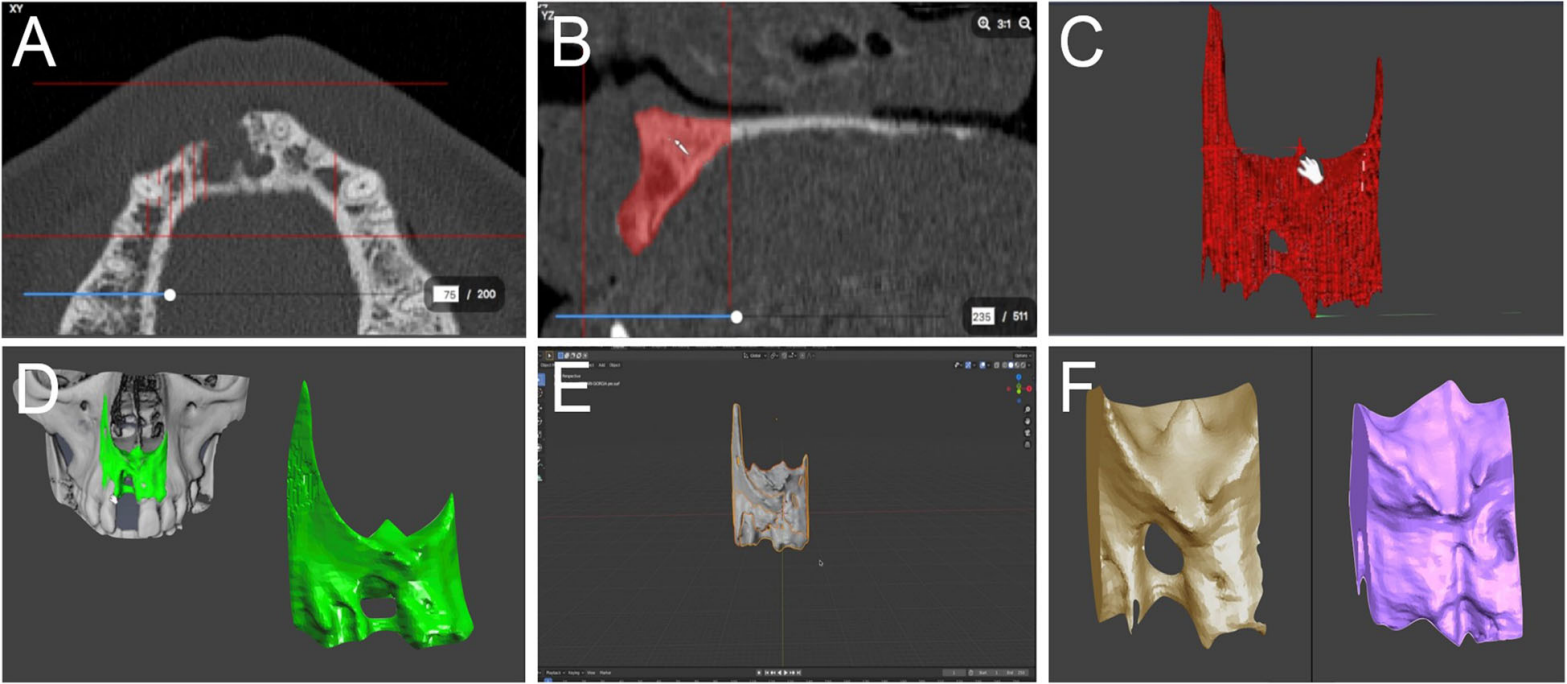
Measurements with dedicated software of bone volume pre- and post-operatively: .dicom files selection (**a**, **b** ,**c**), .stl files creation (**d**), interpolation (**e**), superimposition and volume calculation (**f**)

The .dicom electronic files of pre- and post-operative CBCT were imported to software A to obtain pre- and post- 3D models in .stl files. First, the region of interest was selected using reference points; next, a manual selection based on the grey scale was performed to accurately identify the hard tissues and to exclude the soft tissues; consequently, the selections were interpolated in order to allow the realization of the 3D models. After that, the models were imported into software B, that was used to superimpose the pre- and post- 3D models and to cut them, obtaining the exact volumes of interest. Finally, the differences in volume between the 2 models were measured using the software S, in order to calculate the so-called LBV, which is useful to calculate the RBV, and especially, RR values (Fig. 7).

### Statistical analysis

An Excel data collection form and data management system were used (Excel 2011®; Microsoft Corp., Redmond, WA, USA). All data were entered by a single blinded operator. Prior to be inserted in, all data were evaluated in terms of completeness: logical consistency was verified, and the ranges of quantitative data were computed. Data analysis was performed with a statistical software (STATA/IC software®, StataCorp LLC, College Station, TX, USA). For each variable the mean and standard deviation (SD) were computed and reported.

## Results

In total, 10 consecutive patients, five women and five men, mean age 52 years, showing a localized bone defect in maxilla (*n* = 5) or mandible (n = 5) and in need of bone augmentation were consecutively treated according to the study protocol, from January 2017 to December 2018. The whole digital workflow and the mesh production phases had already been described in a previous study [16]. Five patients had a history of periodontitis or peri-implantitis, two patients had smoking habits (less than 10 cigarettes/die), and no patients had severe systemic or metabolic disease. No dropout occurred during the follow-up.

All surgeries were accomplished without unexpected events. One permanent paresthesia (> 6 months), one temporary paresthesia (< 1 month), one important post-operative hematoma was observed, accounting for a surgical complication rate of 30%. The healing complication rate was 10% due to an early exposure without infection which was in the same patient with the hematoma. According to pseudo-periosteum classification the majority of sites (*n* = 6) were evaulated as belonging to class 1, two sites belonging to class 2, and only one site was classified as class 3. In regard to bone density, most of the sites (*n* = 6) showed medium density, three sites showed hard density, and only one site showed soft density. The mean VBG was 4.5 ± 1.8 mm. The volumetric analysis reported values equal to 984 ± 41 mm^3^, 92 ± 8 mm^3^, and 892 ± 44 mm^3^ for PBV, LBV, and RBV, respectively; as a consequence, the overall RR was 89.04% while pseudo-periosteum represented the remaining 10.96%.

In total, 26 implants were inserted in 10 patients: 14 in the maxilla (7 in the posterior regions and 7 in the pre-maxilla) and 12 in the mandible (10 in the posterior regions and 2 in the interforaminal region). Most of the implants (*n* = 16) achieved a stability between 15 and 35 Ncm; 8 implants higher than 35 Ncm; and only 2 implants less than 15 Ncm. All implants were osseointegrated and were considered successful over time (100% success rate), while the marginal bone loss observed was less than 1.0 mm, 1 year after placement; similarly, prosthetic success rate was 100%. These data are summarized in Tables 1 and 2.

**Table 1:** Data collected at the time of augmentation surgery (T0)

Num. ID	Periodont. Periimplant.	Smoking	Sextant	Ant/Post	Jaw of interest	Defect type	Scarred\Native periosteum	Healing months
1	n	n	II	ant	sup	vert	N	9
2	y	y	VI	post	inf	vert	S	6
3	y	n	I	post	sup	vert	N	9
4	n	y	V	ant	inf	vert	N	9
5	n	n	VI	post	inf	vert	N	6
6	y	n	IV	post	inf	vert	N	6
7	y	n	I	post	sup	vert	N	9
8	y	n	III	post	sup	vert	N	9
9	n	n	IV	post	inf	vert	S	9
10	n	n	II	ant	sup	vert	S	6

Abbreviations: *Periodont.* Periodontitis; *Peri-implant.* Peri-implantitis; *Ant.* Anterior; *Post* Posterior; *Vert.* Vertical

**Table 2:** Data collected at the time of re-entry surgery (T1)

Num. ID	Bone density	Pseudo- periosteum type	Num. of implants	Implant sites	PBV (mm^3^)	LBV (mm^3^)	RBV (mm^3^)	RR %	VBG (mm)	Surgical Complic.	Healing Complic.
1	Medium	2	4	#13, #11, #21, #23	930.0	225.0	705.0	75.8	2.5	.	.
2	Medium	1	3	#45, #46, #47	1430.0	51.0	1379.0	96.4	5.8		.
3	Soft	1	3	#14, #15, #16	960.0	224.0	736.0	76.7	3.9	Permanent Paresthesia	.
4	Hard	2	2	#32, #42	1870.0	17.0	1853.0	99.1	.	.	.
5	Hard	1	2	#45, #47	450.0	33.0	417.0	92.7	3.4	.	.
6	Medium	1	2	#36, #37	840.0	81.0	759.0	90.4	5.3	.	.
7	Medium	1	3	#12, #14, #16	1060.0	46.0	1014.0	95.7	3.5	Temporary Paresthesia	.
8	Medium	2	3	#22, #24, #26	930.0	53.0	877.0	94.3	4.2		.
9	Hard	1	3	#33, #35, #36	780.0	28.0	752.0	96.4	8.5	.	.
10	Medium	3	1	#11	590.0	160.0	430.0	72.9	3.5	Hematoma	Exposure

Abbreviations: *Num.* Number; *PBV* Planned Bone Volume; *LBV* Lacking Bone Volume; *RBV* Regenerated Bone Volume; *RR* Regeneration Rate; *VBG* Vertical Bone Gain; *Complic.* Complications

## Discussion

GBR techniques are widely applied to treat the bone defects of the dental alveolar ridge, and the favorable outcomes of these procedures are founded on an evidence-based rationale [27–32]. The present pilot study described the digital workflow and the clinical outcomes of an innovative GBR approach based on CAD/DMLS customized titanium meshes. Surgical and healing complications were few and did not influence the result of bone augmentation, that achieved about 90% of regenerated bone volume in relation to the planned bone volume. Titanium meshes with their mechanical stiffness and their ability to maintain adequate space underneath allow undisturbed osteogenesis; hence, resulting in a predictable alveolar ridge augmentation in either localized or extended bone defects [8, 9].

In the past, titanium meshes were either bent and adapted directly during the surgery, or cut and shaped on a resin anatomical replica before the surgical intervention [13]. Many authors have used 3D workflows from CBCT data to obtain a physical jawbone model that reproduces an accurate configuration of the defective bone: from this model, a commercial Ti-mesh sheet can be adapted and modeled into a custom-made Ti-mesh. This technique has been described extensively in the last two decades as it can create a containment system for particulate grafts even in case of large reconstructions; however, it requires the pre-bending of the mesh and does not take complete advantage of the CAD/CAM production process [33].

The use of customized CAD/CAM titanium mesh, designed in 3D and fabricated via DMLS can guarantee a practical advantage since the surgeon receives the mesh already in the desired shape, there is no waste of time in adapting it on the site and therefore the intervention is potentially faster, with a reduced risk of infection [16]. In addition, the edges of the mesh are designed so as not to traumatize the soft tissues and avoid exposures [16].

During the development phase of customized devices, the use of dedicated software resulted very effective (i) in performing the virtual augmentation, (ii) in designing a 3D virtual mesh that fits precisely to the residual bone, and (iii) to evaluate the effects of planned surgery superimposing the virtual augmented bone to the native bone profile [16]. The external shape of the mesh should be constructed as round as possible to avoid flap damage; similarly, the surface should be as smooth as possible to avoid bacterial colonization or infection. The thickness can range from 1.0 mm to 0.3 mm, influencing stiffness, malleability, and flexibility of the mesh for better adaptation to the bone deficiencies and to provide the appropriate resistance to mechanical strains. Moreover, the virtual planning must consider the connective tissue that develops between the mesh and the regenerated bone, so-called pseudo-periosteum [27]. As observed in different clinical trials, not all the space under the mesh is filled by newly-formed bone but only about 90% of the planned bone volume is formed, while the rest is represented by connective tissue; for this reason, it is recommended to consider the possibility of over-contouring the mesh during the virtual planning of the bone regeneration. Finally, the pore size and net structure should be carefully considered, as they can influence blood supply and cell invasion in the space underneath the barrier device as well as the amount of newly-formed bone and its remodeling [34, 35].

After completing the CAD design, the. STL files come 3D-printed by mean of a DMLS machine, used to melt a Ti-6AL4V alloy powder in a structure with high mechanical properties and corrosion resistance, low specific weight, and high biocompatibility [35].

The present study reported clinical and radiographic data using different mesh designs in relation to thickness, pore size, net structure, and surface. In absence of severe complications, such as wound dehiscence, early or late exposure with infection, or abscess without exposure, the outcomes of customized meshes allow obtaining an excellent restoration of the original bone deficiency.

Many studies reported encouraging results using different mesh designs: mean VBG ranged from 3.9 mm to 6.5 mm, while exposure rates ranged between 25.0 and 66.0% [14, 36–38]. In this pilot study, the VBG was 4.5 mm and the healing complication rate was 10%, while surgical complications was 30%; moreover, high implant and prosthetic success rates were confirmed at the end of follow-up.

In relation to the complication rates of barrier devices used for reconstructive purposes, a trend in favoring the reduction of the exposure rate of the Ti-mesh has already been reported by some authors, especially in comparison with expanded polytetrafluoroethylene (e-PTFE) membranes [8]. However, Cucchi et al. [39] reported higher complication rates for titanium meshes in comparison to titanium-reinforced d-PTFE membranes. Additionally, a recent systematic review that reported a VBG of about 4.4 mm and 5.2 mm for e-PTFE membranes and titanium meshes, respectively, showed complication rates of 6.9 and 20.0%, respectively [40]. In this regard, the most recent studies pertaining to customized meshes show higher complication rates when compared with conventional Ti meshes; however, the defect type and size, learning curve, patient selection, and other factors play a very important role that may affect the outcomes [14, 37–39] In fact, Sumida et al. [13] aimed to compare customized Ti meshes versus conventional ones in a controlled clinical trial, reporting better results for the experimental group in terms of operative times, exposure/infection rates, and number of fixation screws needed.

In this study a permanent paresthesia in the area of the upper lip was reported from one patient and gives emphasis to the attention that needs to be observed in all phases of the treatment, in particular, in the virtual planning of the mesh which needs to consider the extensive flap management needed to achieve a tension-free closure over the barrier device [41].

Potential disadvantages of customized meshes may be the costs of designing and prototyping the device, and the difficulty in adapting to changes in the intraoperative surgical plan. Another potential disadvantage is the learning curve needed to achieve having confident knowledge of the software [42]. Nevertheless, considering all the drawbacks related to bone augmentation surgery, the technique avoided the majority of complications. In the cases presented in this study, the vertical/horizontal bone regeneration obtained was always adequate in order to finalize the implant-prosthetic treatment. Implant placement was possible in all cases, and the analysis of the augmented bone volume demonstrated significant changes of alveolar ridges, as reported by many authors [14, 37–39].

The limited number of patients treated, the short-term follow-up, and the absence of a control group do not allow drawing strong conclusions about the superiority of this technique in comparison to others, but several advantages can emphasized: the possibility to virtually overview the entire procedure prior to the day of surgery, the shortening of the surgical times, the increased accuracy and stability of the device at the try-in phase, the low incidence of healing complications, the predictability of regenerated bone volumes, and last but not least, the integration of data regarding the planned augmentation in the digital workflow.

## Conclusions

CAD/DLSM customized titanium meshes represent the most recent innovation in the field of bone augmentation. The virtual planning of barrier titanium membranes allows the creation of different designs according to the specific clinical scenario or to clinician’s preferences. The digital workflow and customized designs demonstrated high efficiency to reduce the healing complications, such as early or late exposures, that represent the main issue of concern in vertical ridge augmentation. In our study, all augmented sites were successfully restored with definitive implant-supported fixed partial dentures. Measurements showed an average VBG of 4.5 ± 1.8 mm at surgical re-entry. Surgical and healing complications occurred in 30% and 10% of cases, respectively. Mean values of PBV, LBV, and RBV were 984, 92, and 892 mm^3^, respectively. Moreover, digital analysis allowed to calculate the regenerated bone volume in relation to the planned bone volume, giving the regeneration rate (RR) of 89%. Finally, all 26 implants in augmented sites showed an adequate primary stability and osseointegration and they were successful after functional loading at the 1-year follow-up.

## Data Availability

The complete documentation of all patients enrolled in this study belong to the authors, and are available only upon reasonable request.

## Abbreviations

CAD/CAM: Computer-aided-design/computer-aided-manufacturing
DMLS: Direct Metal Laser Sintering
VBG: Vertical bone gain
PBV: Planned bone volume
LBV: Lacking bone volume
RBV: Regenerated bone volume
RR: Regeneration rate
GBR: Guided bone regeneration
CBCT: Cone-beam-computed-tomography
DICOM: Digital imaging and communication in medicine
STL: Stereolithographic
SLM: Selective laser melting
e-PTFE: expanded polytetrafluoroethylene
